# Supplementary material for: Prognosis value of galectin-3 in patients with dilated cardiomyopathy: a meta-analysis
Source: PeerJ. 2024 Apr 23;12:e17201. doi: 10.7717/peerj.17201 (PMC11048071; doi:10.7717/peerj.17201)
Supplement: Supplemental Information 3 [file peerj-12-17201-s003.docx]

**The rationale for conducting the systematic review / meta-analysis:**

It is important to accurately predict and assess myocardial fibrosis (MF) and adverse cardiovascular events (MACEs) in patients with dilated cardiomyopathy (DCM). Several studies have shown that galectin-3 (gal-3) may be a promising predictor of prognosis in patients with DCM. However, more studies are needed to determine the predictive value of gal-3 in the prognosis of DCM,so we conducted the meta-analysis.

Gal-3 for MACEs in patients with DCM was reported in 7 studies(15, 19-24)that included 945 patients. From the overall analysis, a greater risk of MACEs was observed for an abnormal elevation of gal-3 level(**Figure 4**). The analysis indicated that the increase of gal-3 level was potentially relevant to a greater risk of MACEs in patients with DCM (HR=1.09, 95%CI=1.04-1.14, I^2^ =28.5%,p =0.211).
