# Supplementary figures and images for: Prognosis value of galectin-3 in patients with dilated cardiomyopathy: a meta-analysis
Source: PeerJ. 2024 Apr 23;12:e17201. doi: 10.7717/peerj.17201 (PMC11048071; doi:10.7717/peerj.17201)

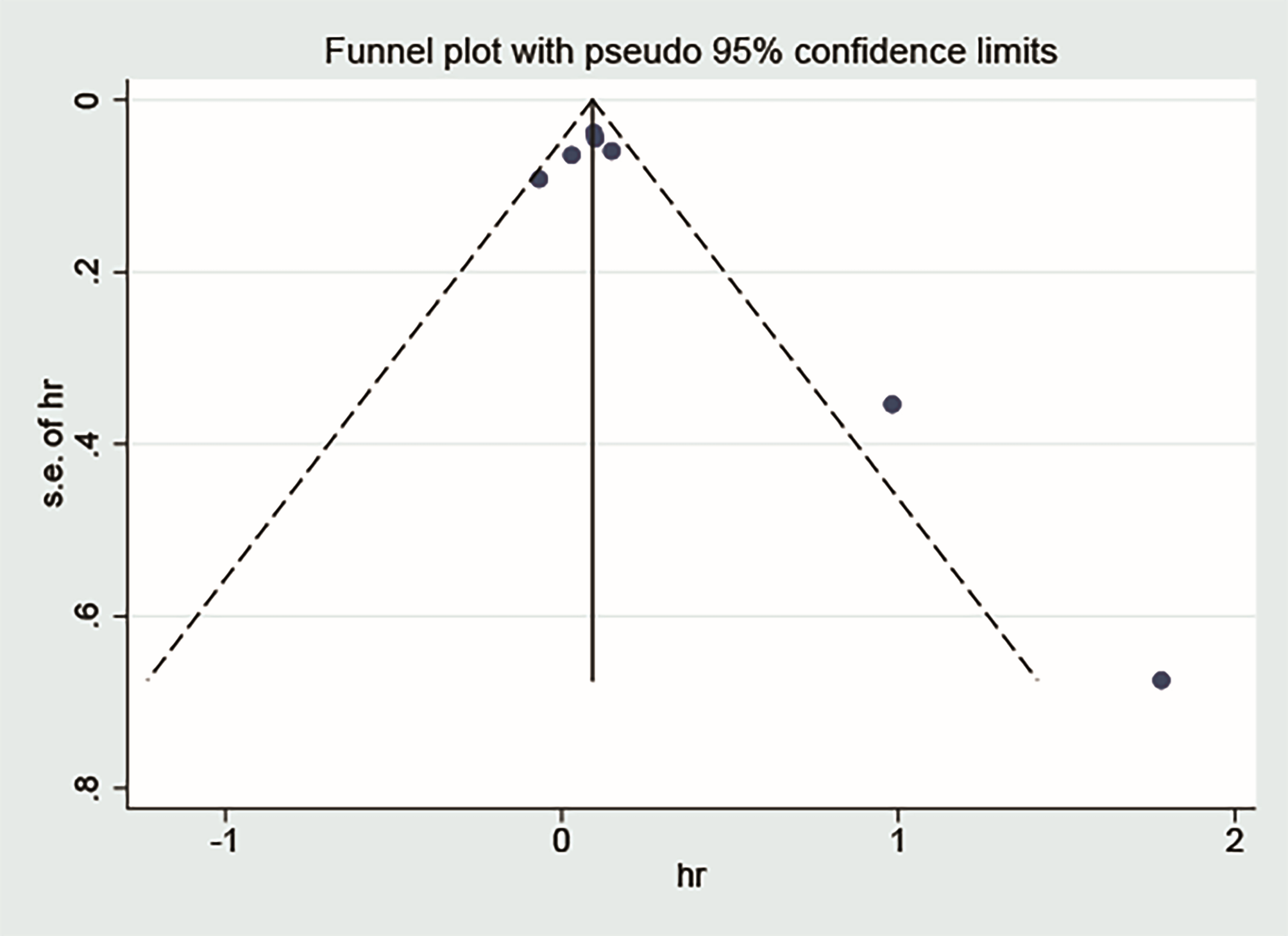

Supplement: Supplemental Information 4 [file peerj-12-17201-s004.png]

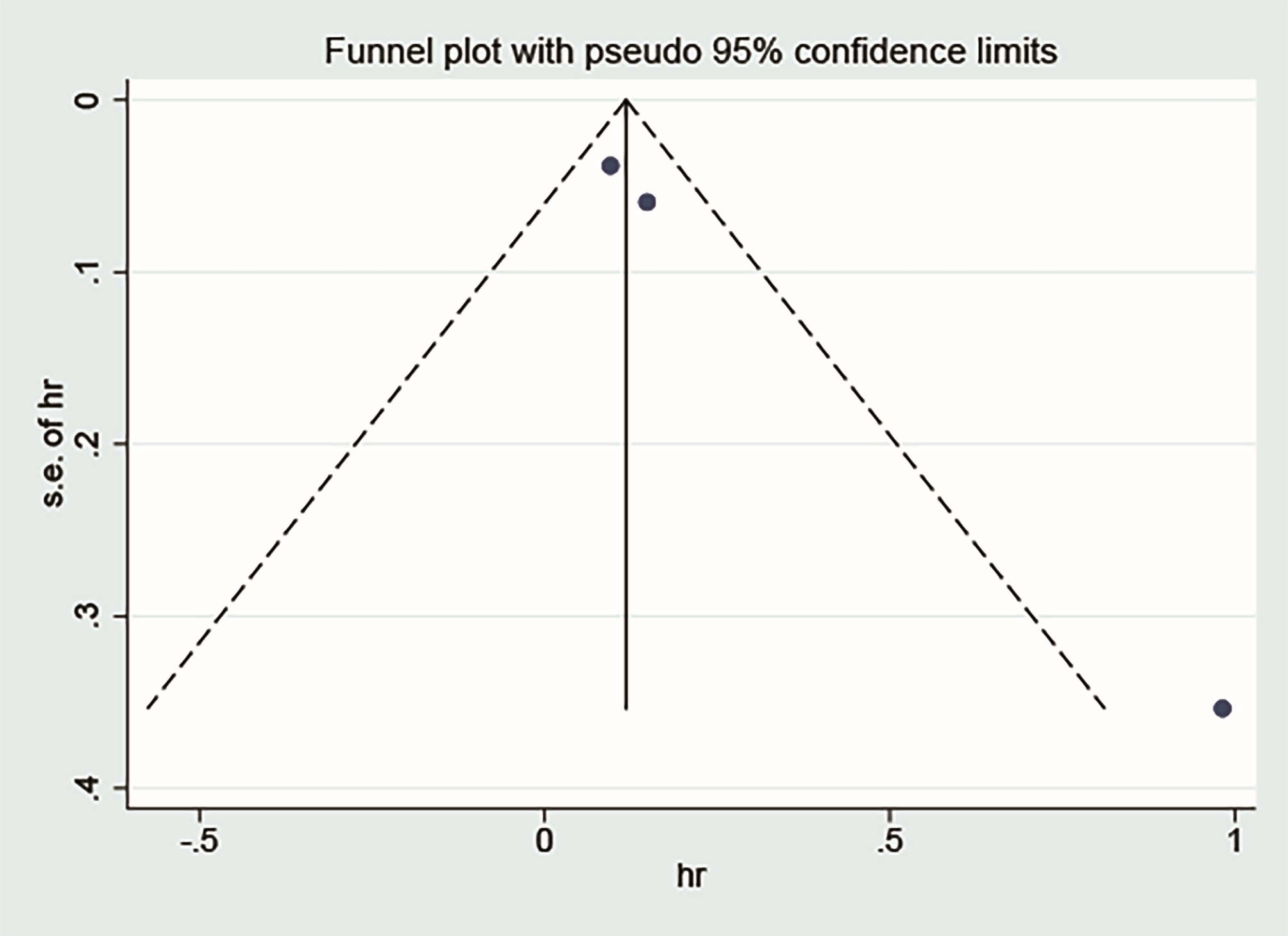

Supplement: Supplemental Information 5 [file peerj-12-17201-s005.png]

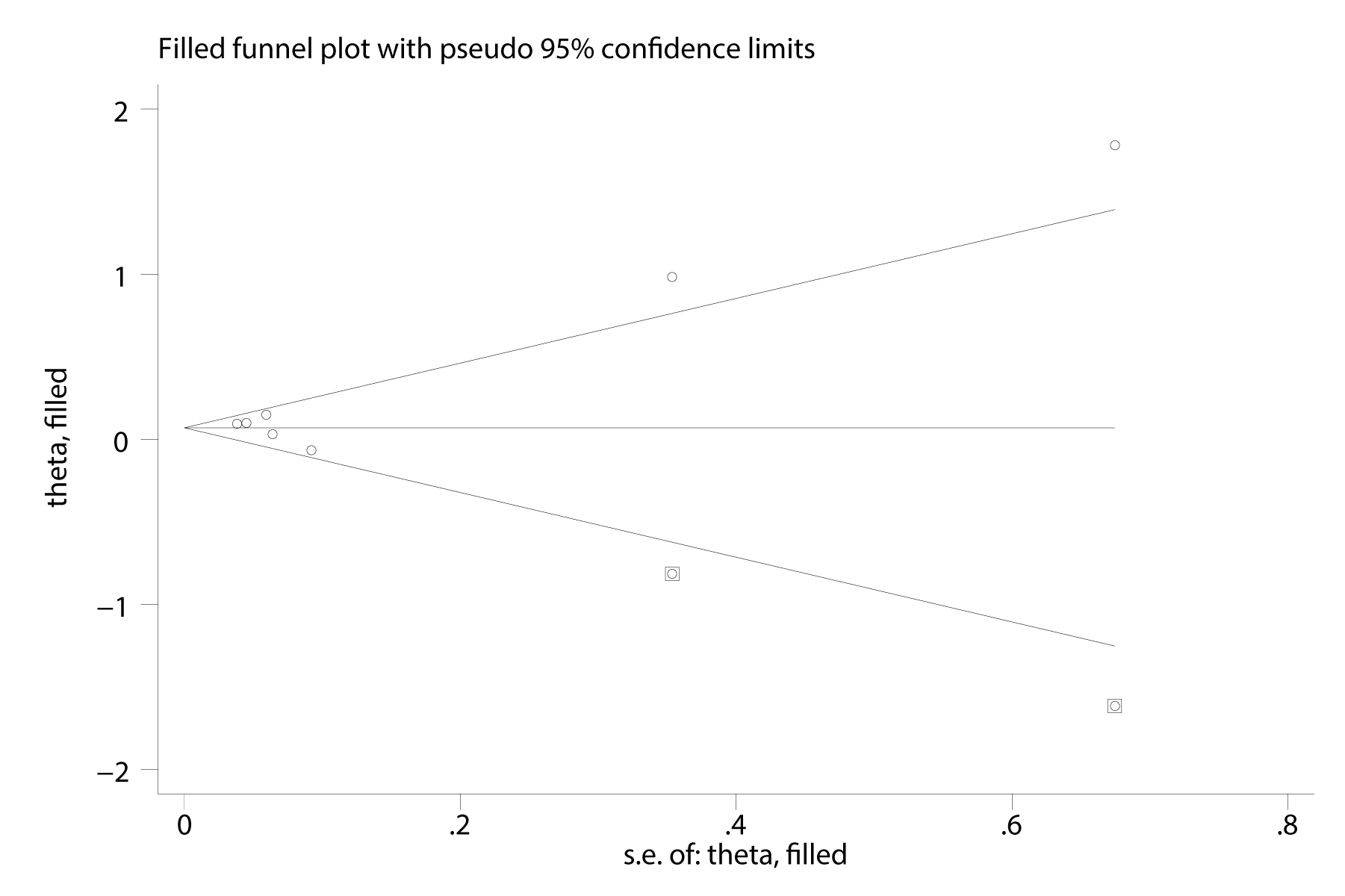

Supplement: Supplemental Information 6 [file peerj-12-17201-s006.png]

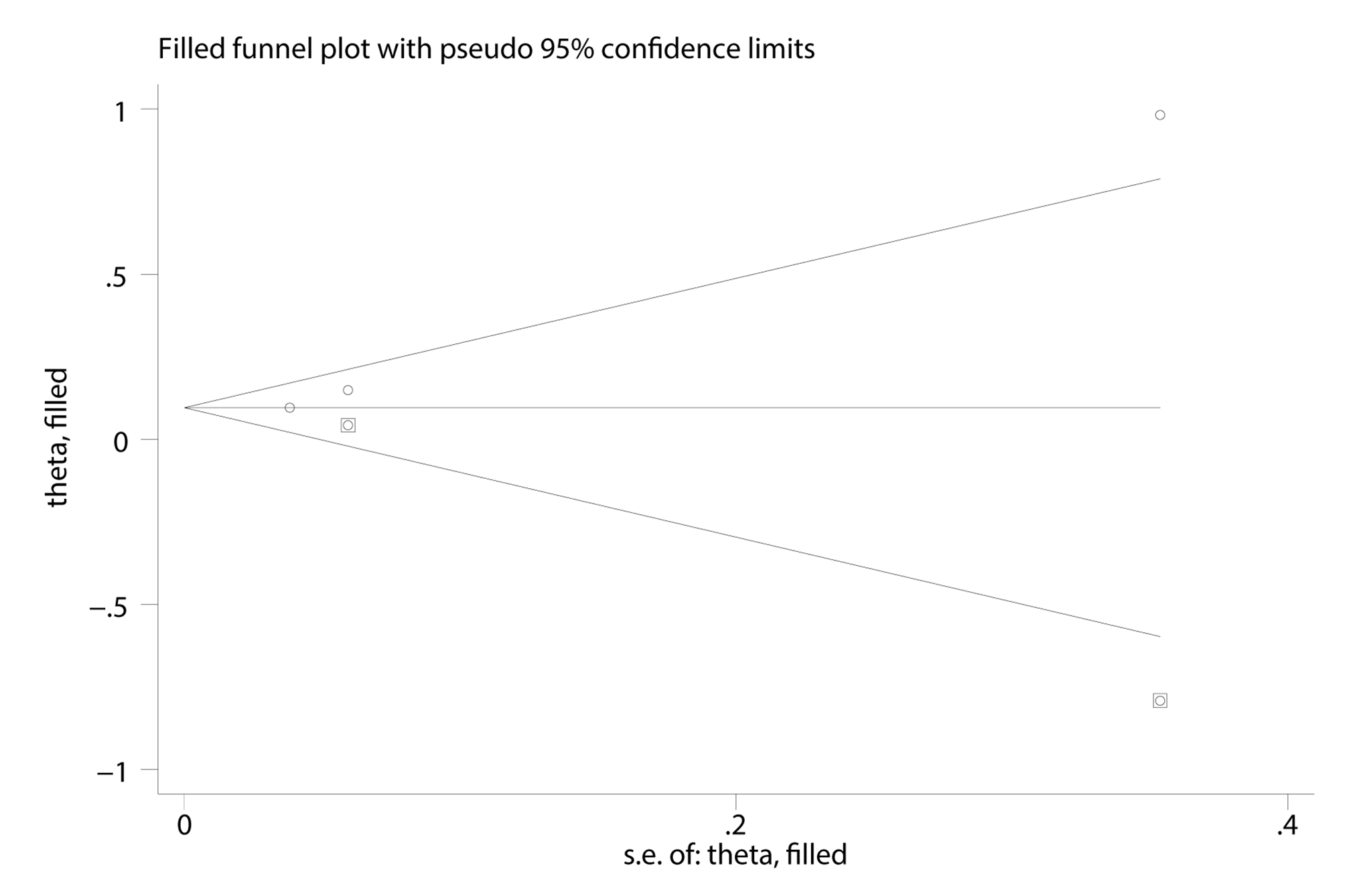

Supplement: Supplemental Information 7 [file peerj-12-17201-s007.png]

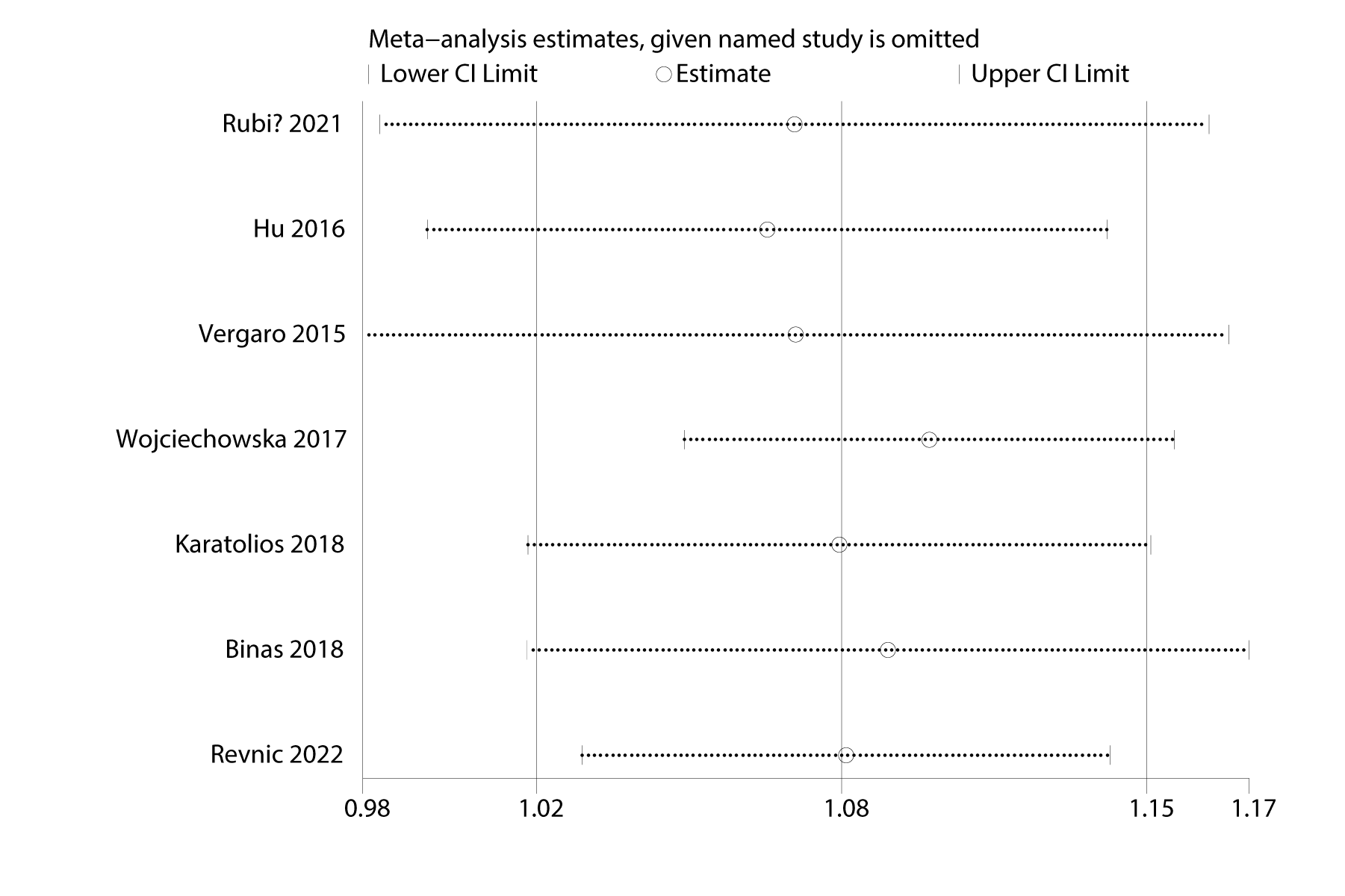

Supplement: Supplemental Information 8 [file peerj-12-17201-s008.png]

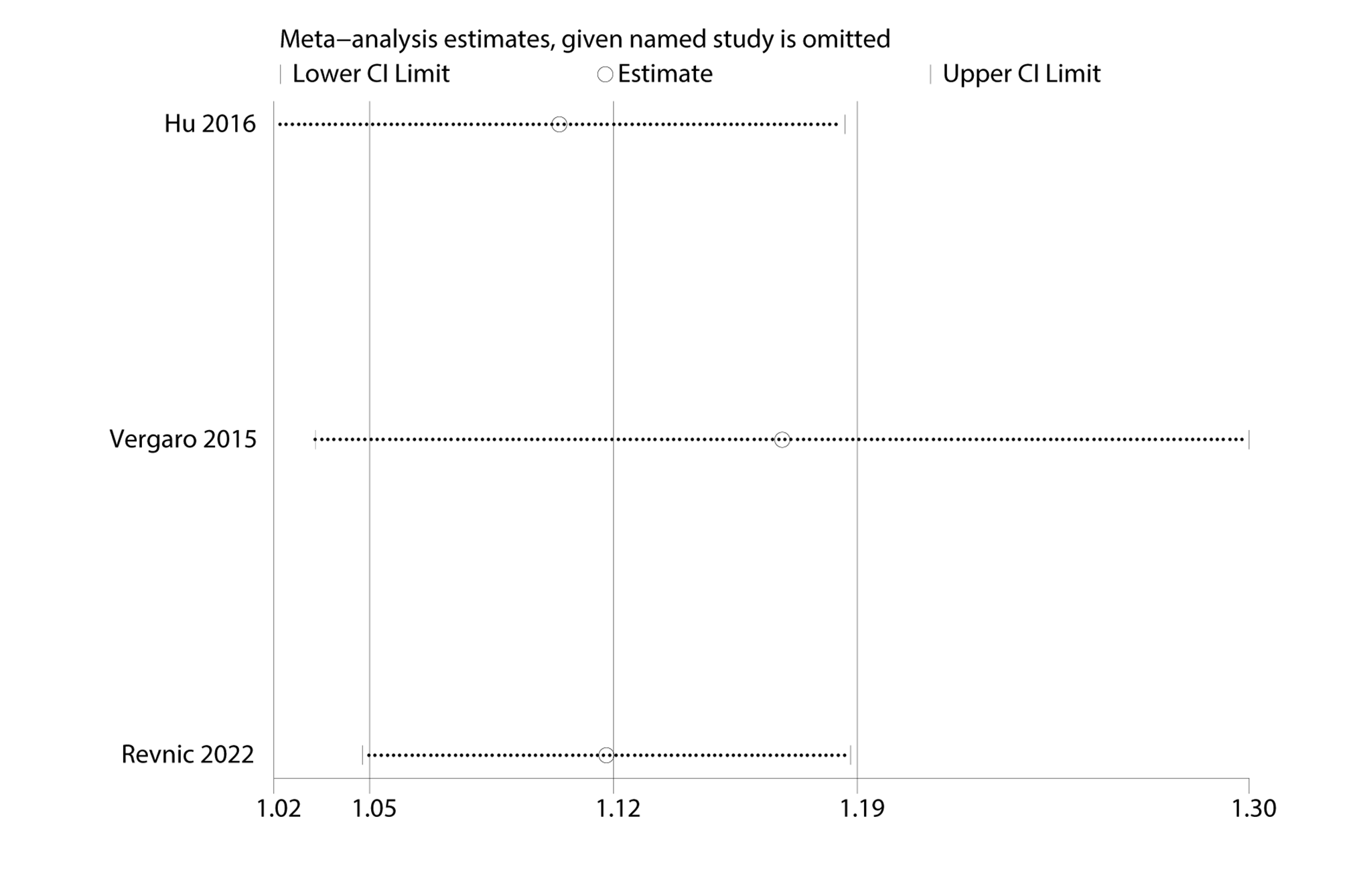

Supplement: Supplemental Information 9 [file peerj-12-17201-s009.png]
