# Supplementary material for: Prognosis value of galectin-3 in patients with dilated cardiomyopathy: a meta-analysis
Source: PeerJ. 2024 Apr 23;12:e17201. doi: 10.7717/peerj.17201 (PMC11048071; doi:10.7717/peerj.17201)
Supplement: Supplemental Information 10 — The participants’ demographic characteristics in the included studies. [file peerj-12-17201-s010.docx]

**Search strategy of PubMed**

| NO. | Search Details | Results |
| --- | --- | --- |
| #5 | (#1 OR #2) AND (#3 OR #4) | 44 |
| #4 | ((((((((((((((((((((Dilated Cardiomyopathies) OR (Dilated Cardiomyopathy)) OR (Familial Idiopathic Cardiomyopathies)) OR (Familial Idiopathic Cardiomyopathy)) OR (Congestive Cardiomyopathy)) OR (Congestive Cardiomyopathies)) OR (Idiopathic Dilated Cardiomyopathies)) OR (Idiopathic Dilated Cardiomyopathy)) OR (Cardiomyopathy, Dilated, LMNA)) OR (Cardiomyopathy, Dilated, Autosomal Recessive)) OR (Cardiomyopathy, Dilated, 1a)) OR (Cardiomyopathy, Dilated, With Conduction Defect 1)) OR (Cardiomyopathy, Dilated, with Conduction Deffect1)) OR (Cardiomyopathy, Dilated, CMD1A)) OR (congestive cardio-myopathy)) OR (congestive CMP)) OR (congestive dilated cardiomyopathy)) OR (congestive myocardiopathies)) OR (congestive myocardiopathy)) OR (dilated cardio-myopathy)) OR (dilated CMP) | 30,953 |
| #3 | "Cardiomyopathy, Dilated"[Mesh] | 17,190 |
| #2 | (((((((((((((((((((((CBP-30) OR (CBP-35)) OR (CBP35)) OR (Epsilon-Binding Protein)) OR (Epsilon Binding Protein)) OR (Galectin-3)) OR (HL-29)) OR (IgE Binding Protein)) OR (IgEBP)) OR (L-29 Lectin)) OR (L 29 Lectin)) OR (L-31)) OR (L-34)) OR (L30 Lectin)) OR (LGALS3)) OR (Mac-2 Antigen)) OR (Mac 2 Antigen)) OR (Macrophage-2 Antigen)) OR (Macrophage 2 Antigen)) OR (Carbohydrate-Binding Protein 35)) OR (Carbohydrate Binding Protein 35)) OR (galectin 3) | 16,323 |
| #1 | "Galectin 3"[Mesh] | 3,459 |

**Search strategy of EMBASE**

| No. | Query | Results |
| --- | --- | --- |
| #5 | (#1 OR #2) AND (#3 OR #4) | 203 |
| #4 | 'dilated cardiomyopathies':ti,ab,kw OR 'dilated cardiomyopathy':ti,ab,kw OR 'familial idiopathic cardiomyopathies':ti,ab,kw OR 'familial idiopathic cardiomyopathy':ti,ab,kw OR 'congestive cardiomyopathy':ti,ab,kw OR 'congestive cardiomyopathies':ti,ab,kw OR 'idiopathic dilated cardiomyopathies':ti,ab,kw OR 'idiopathic dilated cardiomyopathy':ti,ab,kw OR 'cardiomyopathy, dilated, lmna':ti,ab,kw OR 'cardiomyopathy, dilated, autosomal recessive':ti,ab,kw OR 'cardiomyopathy, dilated, 1a':ti,ab,kw OR 'cardiomyopathy, dilated, with conduction defect 1':ti,ab,kw OR 'cardiomyopathy, dilated, with conduction deffect1':ti,ab,kw OR 'cardiomyopathy, dilated, cmd1a':ti,ab,kw OR 'congestive cardio-myopathy':ti,ab,kw OR 'congestive cmp':ti,ab,kw OR 'congestive dilated cardiomyopathy':ti,ab,kw OR 'congestive myocardiopathies':ti,ab,kw OR 'congestive myocardiopathy':ti,ab,kw OR 'dilated cardio-myopathy':ti,ab,kw OR 'dilated cmp':ti,ab,kw | 33441 |
| #3 | 'dilated cardiomyopathy'/exp | 86331 |
| #2 | 'cbp-30':ti,ab,kw OR 'cbp-35':ti,ab,kw OR 'cbp35':ti,ab,kw OR 'epsilon-binding protein':ti,ab,kw OR 'epsilon binding protein':ti,ab,kw OR 'galectin-3':ti,ab,kw OR 'hl-29':ti,ab,kw OR 'ige binding protein':ti,ab,kw OR 'igebp':ti,ab,kw OR 'l-29 lectin':ti,ab,kw OR 'l 29 lectin':ti,ab,kw OR 'l-31':ti,ab,kw OR 'l-34':ti,ab,kw OR 'l30 lectin':ti,ab,kw OR 'lgals3':ti,ab,kw OR 'mac-2 antigen':ti,ab,kw OR 'mac 2 antigen':ti,ab,kw OR 'macrophage-2 antigen':ti,ab,kw OR 'macrophage 2 antigen':ti,ab,kw OR 'carbohydrate-binding protein 35':ti,ab,kw OR 'carbohydrate binding protein 35':ti,ab,kw OR 'galectin 3':ti,ab,kw | 9644 |
| #1 | 'galectin 3'/exp | 9877 |

**Search strategy of Cochrane Library**

| NO. | Search deatiles | Hits |
| --- | --- | --- |
| #1 | MeSH descriptor: [Galectin 3] explode all trees | 50 |
| #2 | MeSH descriptor: [Cardiomyopathy, Dilated] explode all trees | 608 |
| #3 | (CBP-30):ti,ab,kw OR (CBP-35):ti,ab,kw OR (CBP35):ti,ab,kw OR (Epsilon-Binding Protein):ti,ab,kw OR (Epsilon Binding Protein):ti,ab,kw OR (Galectin-3):ti,ab,kw OR (HL-29):ti,ab,kw OR (IgE Binding Protein):ti,ab,kw OR (IgEBP):ti,ab,kw OR (L-29 Lectin):ti,ab,kw OR (L 29 Lectin):ti,ab,kw OR (L-31):ti,ab,kw OR (L-34):ti,ab,kw OR (L30 Lectin):ti,ab,kw OR (LGALS3):ti,ab,kw OR (Mac-2 Antigen):ti,ab,kw OR (Mac 2 Antigen):ti,ab,kw OR (Macrophage-2 Antigen):ti,ab,kw OR (Macrophage 2 Antigen):ti,ab,kw OR (Carbohydrate-Binding Protein 35):ti,ab,kw OR (Carbohydrate Binding Protein 35):ti,ab,kw OR (galectin 3):ti,ab,kw | 1038 |
| #4 | (Dilated Cardiomyopathies):ti,ab,kw OR (Dilated Cardiomyopathy):ti,ab,kw OR (Familial Idiopathic Cardiomyopathies):ti,ab,kw OR (Familial Idiopathic Cardiomyopathy):ti,ab,kw OR (Congestive Cardiomyopathy):ti,ab,kw OR (Congestive Cardiomyopathies):ti,ab,kw OR (Idiopathic Dilated Cardiomyopathies):ti,ab,kw OR (Idiopathic Dilated Cardiomyopathy):ti,ab,kw OR (Cardiomyopathy, Dilated, LMNA):ti,ab,kw OR (Cardiomyopathy, Dilated, Autosomal Recessive):ti,ab,kw OR (Cardiomyopathy, Dilated, 1a):ti,ab,kw OR (Cardiomyopathy, Dilated, With Conduction Defect 1):ti,ab,kw OR (Cardiomyopathy, Dilated, with Conduction Deffect1):ti,ab,kw OR (Cardiomyopathy, Dilated, CMD1A):ti,ab,kw OR (congestive cardio-myopathy):ti,ab,kw OR (congestive CMP):ti,ab,kw OR (congestive dilated cardiomyopathy):ti,ab,kw OR (congestive myocardiopathies):ti,ab,kw OR (congestive myocardiopathy):ti,ab,kw OR (dilated cardio-myopathy):ti,ab,kw OR (dilated CMP):ti,ab,kw | 1592 |
| #5 | (#1 or #3) and (#2 or #4) | 10 |

**Search strategy of web of science**

| NO. | Search deatiles | Results |
| --- | --- | --- |
| #1 | ((((((((((((((((((((TS=(CBP-30) OR TS=(CBP-35)) OR TS=(CBP35)) OR TS=(Epsilon-Binding Protein)) OR TS=(Epsilon Binding Protein)) OR TS=(Galectin-3)) OR TS=(HL-29)) OR TS=(IgE Binding Protein)) OR TS=(IgEBP)) OR TS=(L-29 Lectin)) OR TS=(L 29 Lectin)) OR TS=(L-31)) OR TS=(L-34)) OR TS=(L30 Lectin)) OR TS=(LGALS3)) OR TS=(Mac-2 Antigen)) OR TS=(Mac 2 Antigen)) OR TS=(Macrophage-2 Antigen)) OR TS=(Macrophage 2 Antigen)) OR TS=(Carbohydrate-Binding Protein 35)) OR TS=(Carbohydrate Binding Protein 35)) OR TS=(galectin 3) | 36027 |
| #2 | (((((((((((((((((((TS=(Dilated Cardiomyopathies) OR TS=(Dilated Cardiomyopathy)) OR TS=(Familial Idiopathic Cardiomyopathies)) OR TS=(Familial Idiopathic Cardiomyopathy)) OR TS=(Congestive Cardiomyopathy)) OR TS=(Congestive Cardiomyopathies)) OR TS=(Idiopathic Dilated Cardiomyopathies)) OR TS=(Idiopathic Dilated Cardiomyopathy)) OR TS=(Cardiomyopathy, Dilated, LMNA)) OR TS=(Cardiomyopathy, Dilated, Autosomal Recessive)) OR TS=(Cardiomyopathy, Dilated, 1a)) OR TS=(Cardiomyopathy, Dilated, With Conduction Defect 1)) OR TS=(Cardiomyopathy, Dilated, with Conduction Deffect1)) OR TS=(Cardiomyopathy, Dilated, CMD1A)) OR TS=(congestive cardio-myopathy)) OR TS=(congestive CMP)) OR TS=(congestive dilated cardiomyopathy)) OR TS=(congestive myocardiopathies)) OR TS=(congestive myocardiopathy)) OR TS=(dilated cardio-myopathy)) OR TS=(dilated CMP) | 39556 |
| #3 | #2 AND #1 | 98 |
