# Supplementary material for: Prognosis value of galectin-3 in patients with dilated cardiomyopathy: a meta-analysis
Source: PeerJ. 2024 Apr 23;12:e17201. doi: 10.7717/peerj.17201 (PMC11048071; doi:10.7717/peerj.17201)
Supplement: Supplemental Information 11 — There may be publication bias in gal-3 in LGE(+) vs LGE(-) group. [file peerj-12-17201-s011.docx]

**Supplementary Table 2. Publication bias and heterogeneity of summarized outcomes**

| **Outcomes** | **Publication bias** | |
| --- | --- | --- |
|  | **Begg (*P* value)** | **Egger (*P* value)** |
| ***gal-3 and cardiovascular adverse events in patients with dilated cardiomyopathy*** | 0.453 | 0.146 |
| ***gal-3 in patients with dilated cardiomyopathy and non-dilated disease*** | 0.117 | 0.022 |

Abbreviation: NA, not available.
